# Molecular Cloning and Functional Identification of the Antimicrobial Peptide Gene Ctri9594 from the Venom of the Scorpion *Chaerilus tricostatus*

**DOI:** 10.3390/antibiotics10080896

**Published:** 2021-07-23

**Authors:** Dangui He, Zhijian Cao, Ruhong Zhang, Wenhua Li

**Affiliations:** 1 Hubei Key Laboratory of Cell Homeostasis, College of Life Sciences, Wuhan University, Wuhan 430072, China; 2018302040144@whu.edu.cn; 2 State Key Laboratory of Virology, College of Life Sciences, Wuhan University, Wuhan 430072, China; zjcao@whu.edu.cn; 3 Renmin Hospital of Wuhan University, Wuhan 430200, China

**Keywords:** *Chaerilus tricostatus*, scorpion venom peptide, antimicrobial peptide, Ctri9594

## Abstract

Scorpion venom is a mixture of bioactive peptides, among which neurotoxins and antimicrobial peptides serve especially vital functions. Scorpion venom peptides in Buthidae species have been well described, but toxic peptides from non-Buthidae species have been under-investigated. Here, an antimicrobial peptide gene, Ctri9594, was cloned and functionally identified from the venom of the scorpion *Chaerilus tricostatus.* The precursor nucleotide sequence of Ctri9594 is 199 nt in length, including a 43 nt 5′ UTR, 115 nt 3′ UTR and 210 nt ORF. The ORF encodes 69 amino acid residues, containing a 21 aa signal peptide, 14 aa mature peptide, 3 aa C-terminal posttranslational processing signal and 31 aa propeptide. Multiple sequence alignment and evolutionary analyses show that Ctri9594 is an antimicrobial peptide in scorpion venom. The mature peptide of Ctri9594 was chemically synthesized with a purity greater than 95% and a molecular mass of 1484.4 Da. Minimum inhibitory concentrations (MICs) indicate that the synthesized mature peptide of Ctri9594 has inhibitory activity against Gram-positive bacteria (*Bacillus thuringensis*, *Bacillus subtilis*, *Staphylococcus aureus* and *Micrococcus luteus*) but not Gram-negative bacteria (*Escherichia coli* and *Pseudomonas aeruginosa*) or a fungus (*Candida albicans*). The antimicrobial mechanism of Ctri9594 is inferred to be related to its amphiphilic α-helix structure.

## 1. Introduction

The venom produced by scorpions to kill prey and defend against natural enemies is rich in bioactive components. The active components of scorpion venom include enzymes, peptides, nucleotides, biogenic amines and other unknown components, among which enzymes and peptides perform the most important activities [1]. Currently, neurotoxins are considered to be the most important type of scorpion venom component. These compounds mainly act on sodium, potassium, chloride or calcium channels [2]. Antimicrobial peptides are the other typical class of scorpion venom peptides that have been extensively researched [3].

Antimicrobial peptides isolated from scorpion venom can be divided into the following three classes. The members of the first class include scorpine and its homologous peptides. Scorpine, which is isolated from the scorpion *Pandinus imperator*, has antibacterial and antimalarial activities [4]. Peptides homologous to scorpine have been found in other scorpion species [5,6]. To date, this type of peptide has only been found in non-Buthidae scorpion species. These antimicrobial peptides are composed of 75–78 amino acid residues, with an N-terminal region similar to cecropins, with an amphiphilic α-helix structure and a C-terminal region containing a CSαβ motif. Long non-disulfide antimicrobial peptides are the second class and mainly include parabutoporin, pandinin-1, opistoporins and hadurin [7]. These antimicrobial peptides are composed of 41-44 amino acid residues, and their precursors include signal peptides, mature peptides and C-terminal propeptides. They are rich in basic amino acid residues and can form amphiphilic α-helical structures. Short non-disulfide antimicrobial peptides are the third group, including BmKn2 and IsCT, among others [8,9]. These members generally consist of 13-24 amino acid residues. Their precursor organization and structure (signal peptide, mature peptide and C-terminal propeptide) are the same as those of the second class of AMPs.

Most of the previous reports on scorpion venom peptides have focused on a few species of the family Buthidae, and there has been little investigation on the venoms of non-Buthidae species. The venom peptides of non-Buthidae species also have unique research and application value [10], such as the analysis of toxin evolution and diversity and the discovery of new toxin functions. The scorpion *Chaerilus tricostatus* is one species of the family Chaerilidae and is mainly distributed in Tibet in China and Assam in India [11].

Transcriptomic analysis of the scorpion *Chaerilus tricostatus* has been performed, but, to date, only a few of its venom peptides have been characterized [12,13,14,15]. In this study, a new nucleotide precursor encoding an antimicrobial peptide was isolated and characterized from the venom gland cDNA library of the scorpion *Chaerilus tricostatus.* The chemically synthesized mature peptide was confirmed to have antimicrobial activity. The inhibitory mechanism of this new venom peptide against bacteria is also discussed.

## 2. Results

### 2.1. Cloning and Characterization of a Precursor Nucleotide Sequence Encoding an AMP from cDNA Library of the Venom Gland of the Scorpion Chaerilus tricostatus

**Figure 1:**
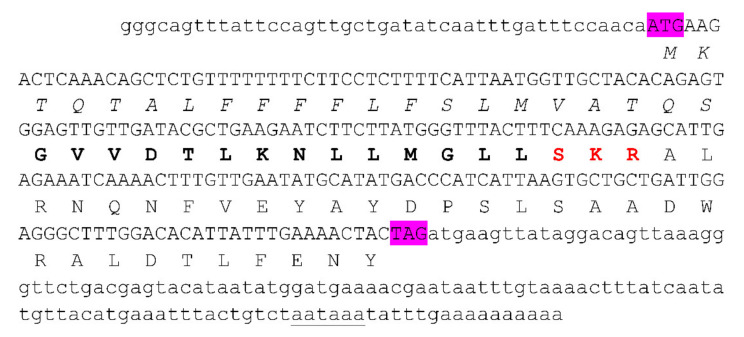
Precursor nucleotide sequences and the deduced amino acid sequences of the scorpion venom peptide Ctri9594 from *Chaerilus tricostatus*. The sequences of 5′ UTR and 3′ UTR are lowercase letters. The sequences of the open reading frame (ORF) are capital letters. The initial codon ATG and the terminal codon TAG are highlighted in purple. A potential polyadenylation signal aataaa is underlined. The predicted amino acid sequences are shown below the corresponding nucleotide sequences. The signal peptide residues are italicized. The mature peptide residues are highlighted in bold. The post-translational processing signal (SKR) is in red.

The cDNA library of the venom gland of the scorpion *Chaerilus tricostatus* was previously constructed [12]. After a large-scale screening, sequencing and analysis of this venom gland cDNA library, a precursor nucleotide sequence encoding a new antimicrobial peptide, Ctri9594, was cloned and characterized. The full-length cDNA of Ctri9594 is 199 nt and consists of three regions: 5′ UTR, open reading frame (ORF) and 3′ UTR. The lengths of 5′ and 3′ UTRs are 43 and 115 nt, respectively, and the open reading frame is 210 nt, encoding a precursor composed of 69 amino acid residues (Figure 1). As shown in Figure 1, the polyadenylation signal aataaa is located 6 nt upstream of the poly-A tail. 

### 2.2. Precursor Amino Acid Sequence Analysis of the Antimicrobial Peptide Ctri9594 from the Venom of the Scorpion Chaerilus tricostatus

Analysis using the SignalP 5.0 software program (http://www.cbs.dtu.dk/services/SignalP/; accessed on: 1 April 2019) showed that the precursor of Ctri9594 has a signal peptide composed of 21 amino acid residues at the N-terminus, followed by a mature peptide composed of 14 amino acid residues and a C-terminal propeptide composed of 31 amino acid residues. The N-terminus of the propeptide contains a processing signal sequence composed of the three amino acid residues Ser-Lys-Arg (SKR), which is located at amino acids 36-38 of the whole precursor sequence (Figure 1). In general, the excision of the C-terminal propeptide results in the amidation of the C-terminal amino acid residue of the mature peptide [16]. This organization and structure of the precursor amino acid sequence are common in antimicrobial peptides in other scorpion species, such as the scorpion IsCT gene family, and other animals, such as the frog temporin gene family [17,18].

### 2.3. Comparison and Evolutionary Analysis of Ctri9594 and Its Homologs

**Figure 2:**
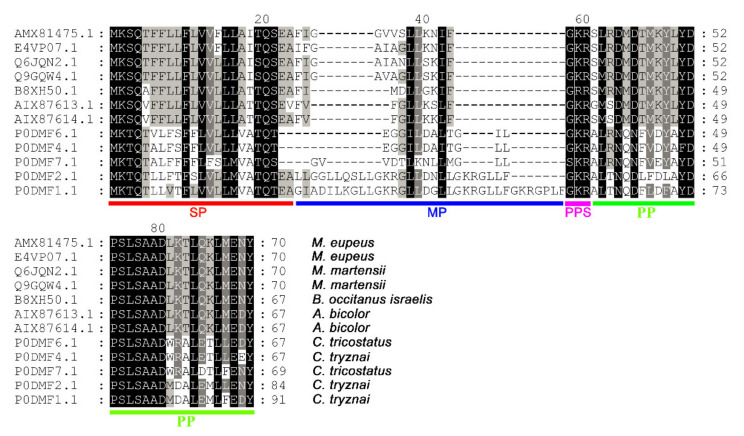
Multiple sequence alignments of the scorpion venom peptide Ctri9594 with its homologs. Sequence alignments of Ctri9594 with similar scorpion venom peptides were carried out by GeneDoc. The residues in black are highly conserved sites, the residues represented by dashes are less conserved sites and the residues without background color are highly various sites. AMX81475.1 and E4VP07.1 are from *Mesobuthus eupeus*. Q6JQN2.1 and Q9GQW4.1 are from *Mesobuthus martensii*. B8XH50.1 is from *Buthus occitanus israelis*. AIX87613.1 and AIX87614.1 are from *Androctonus bicolor*. P0DMF6.1 and P0DMF7.1 (corresponding to Ctri9594) are from *Chaerilus tricostatus*. P0DMF1.1, P0DMF2.1 and P0DMF4.1 are from *Chaerilus tryznai*. SP, signal peptide. MP, mature peptide. PPS, posttranslational processing signal. PP, propeptide.

A series of scorpion antimicrobial peptide precursors with similarities to Ctri9594 were obtained by sequence alignment in GenBank via the NCBI blast program, as shown in Figure 2. Importantly, their mature peptide (MP) regions have low homology. On the contrary, both the N-terminal signal peptide (SP) and C-terminal propeptide (PP) sequences of Ctri9594 show high similarity to 11 other scorpion antimicrobial peptide members. Moreover, the precursors of these peptides encoded by cDNAs share a common structure with short cationic antimicrobial peptides from other scorpion species and animals such as bees, spiders and frogs, all of which have a precursor organization characterized by a signal peptide, mature peptide, post-translational processing signal and propeptide. Therefore, Ctri9594 is presumed to be a new short cationic antimicrobial peptide from the venom of the scorpion *Chaerilus tricostatus*.

**Figure 3:**
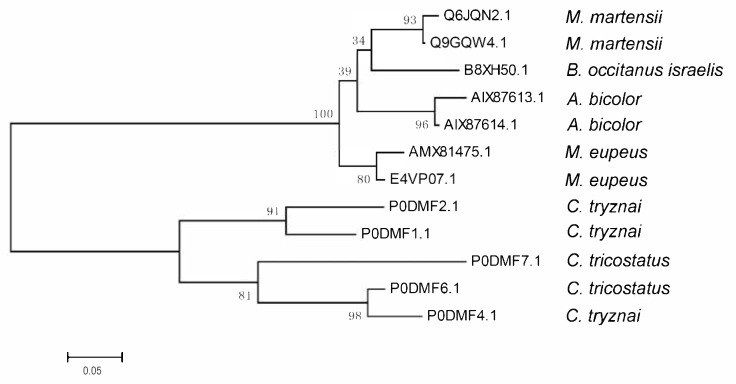
Evolutionary relationship of the scorpion venom peptide Ctri9594 with its homologs. The evolutionary relationship of the scorpion venom peptide Ctri9594 with its homologs was inferred using the neighbor-joining method [19]. The bootstrap consensus tree inferred from 10,000 replicates [20] is taken to represent the evolutionary relationship. Evolutionary analyses were conducted in MEGA7 [21].

Because their mature peptide regions have low homology, but the N-terminal signal peptide (SP) and C-terminal propeptide (PP) regions show high similarity to each other, the evolutionary relationships of Ctri9594 and 11 other scorpion homologs were constructed by using their precursor amino acid sequences. The neighbor-joining tree indicates that 12 scorpion antimicrobial peptide members are clustered into two groups (Figure 3). The first group includes seven members from four scorpion species of the family Buthidae: *Mesobuthus eupeus*, *Mesobuthus martensii*, *Buthus occitanus israelis* and *Androctonus bicolor*. The second group contains five members from two scorpion species, *Chaerilus tricostatus* and *Chaerilus tryznai*, both belonging to the family Chaerilidae (Figure 3). This evolutionary relationship implies that the ancestral species of both the Buthidae and Chaerilidae families had the ancestral gene encoding this kind of antimicrobial peptide. In the family Buthidae, three scorpion species each contain two closer members, possibly indicating that the common ancestral species of the family Buthidae had two homologous genes. *Buthus occitanus israelis* presumably has a second homolog yet to be identified. 

### 2.4. Structural Analysis of the Chemically Synthesized Ctri9594 Mature Peptide

**Figure 4:**
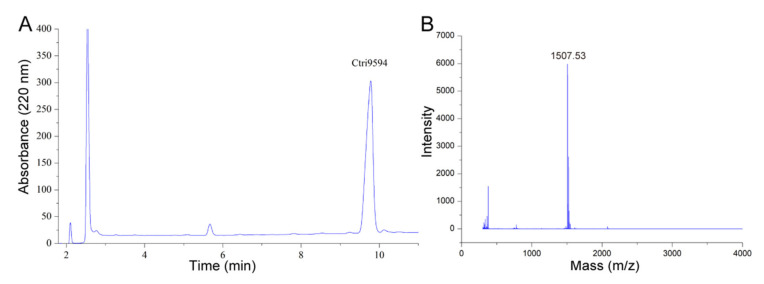
RP-HPLC and mass spectrometry analyses of the chemically synthesized Ctri9594 mature peptide. (**A**) RP-HPLC profile of the chemically synthesized Ctri9594 mature peptide. The fraction containing the chemically synthesized Ctri9594 mature peptide is indicated. The purity of the chemically synthesized Ctri9594 peptide is more than 95%. (**B**) Mass spectrometry analysis of the chemically synthesized Ctri9594 mature peptide measured by MALDI-TOF-MS. The measured molecular mass of the chemically synthesized Ctri9594 mature peptide is 1484.53 Da, corresponding well to the theoretical calculated molecular mass of 1484.85 Da.

As shown in Figure 4, the purity and molecular mass of the chemical synthetic peptide Ctri9594 were determined by RP-HPLC and mass spectrometry, respectively. The results show that the purity was more than 95%. The *m*/*z* of the chemical synthetic peptide Ctri9594 was determined to be 1507.53, indicating that the peptide Ctri9594 was sodium salt and the measured molecular mass of Ctri9594 was 1484.53 Da. This measured molecular mass is very close to its predicted molecular mass of 1484.85 Da.

**Figure 5:**
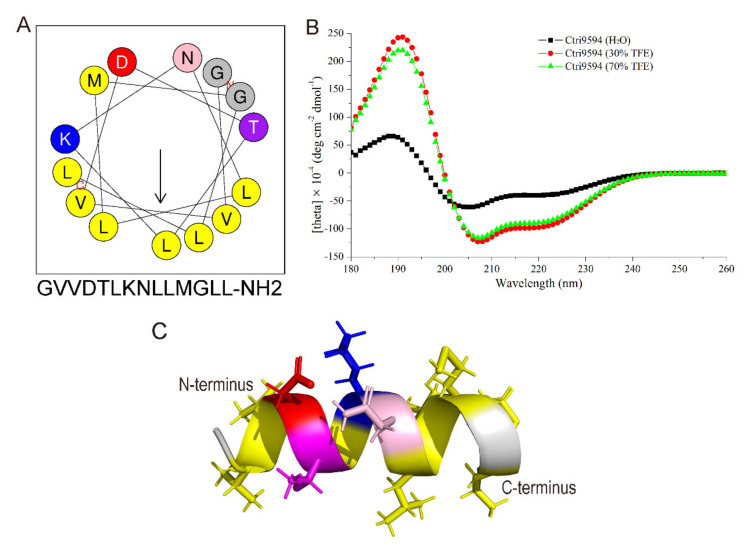
Structure analysis of Ctri9594 peptide. (**A**) Helical wheel diagram of the peptide Ctri9594 determined by the Heliquest method. The representation of the peptide Ctri9594 as a helical wheel shows the hydrophilic face and hydrophobic face. (**B**) CD spectra of the peptide Ctri9594 (100 mg/mL) in H_2_O alone or with 30 or 70% aqueous TFE. (**C**) Molecular modeling of three-dimensional structure of the peptide Ctri9594. Amino acid residues Leu (L), Val (V) and Met (M) are in yellow. Amino acid residue Asp (D) is in red. Amino acid residue Lys (K) is in blue. Amino acid residue Thr (T) is in purple. Amino acid residue Asn (N) is in pink. Amino acid residue Gly (G) is in gray.

As shown in Figure 5A, the secondary structure of Ctri9594 predicted by the Heliquest website is a typical amphipathic molecule. The helix structure is divided into two different sides. One side is the hydrophobic surface composed of hydrophobic amino acid residues, and the other side is the hydrophilic surface composed of hydrophilic amino acid residues. There are two lysine residues with a positive charge on the hydrophilic surface. The predicted result shows that Ctri9594 is an amphiphilic α-helix peptide with two positive charges. The circular dichroism experiment further confirmed the predicted α-helix structure (Figure 5B). From the curve in the figure, Ctri9594 can form a distinct α-helix structure in the presence of TFE (30% or 70%), but it adopts a random structure in ultrapure water (H_2_O). Furthermore, the three-dimensional structure of the peptide Ctri9594 was modeled using the website I-TASSER (https://zhanglab.dcmb.med.umich.edu/I-TASSER/; accessed on: 10 January 2009) and displayed with PDB Viewer software. The modeling result indicates that the peptide Ctri9594 is a typical α-helix structure (Figure 5C). The hydrophobic amino acid residues Leu (L) and Val (V) (displayed in yellow) are located on the same surface, and the hydrophilic amino acid residues Lys (K), Thr (T), Asp (D) and Asn (N) appear on the opposite surface. These results show that the peptide Ctri9594 can form an amphiphilic α-helix structure under suitable membrane conditions, suggesting its bactericidal activity.

### 2.5. Antimicrobial Activity of the Chemically Synthesized Ctri9594 Mature Peptide

In order to further investigate the antimicrobial activity, the minimal inhibitory concentrations (MICs) of the chemically synthesized Ctri9594 mature peptide against standard strains of Gram-negative bacteria, Gram-positive bacteria and fungus were determined by the microdilution method. As shown in Table 1, Ctri9594 had inhibitory effects on *Bacillus thuringensis* AB92037, *Bacillus subtilis* AB91021, *Staphylococcus aureus* AB94004 and *Micrococcus luteus* AB93113, but it had no inhibitory activity against *Escherichia coli* AB94012, *Pseudomonas aeruginosa* AB93066 or *Candida albicans* AY93025 at a concentration of 100 μg/mL. The minimum inhibitory concentration of Ctri9594 against *Bacillus thuringensis* AB92037, *Bacillus subtilis* AB91021 and *Staphylococcus aureus* AB94004 was 25 μg/mL, and its MIC against *Micrococcus luteus* AB93113 was 12.5 μg/mL (Table 1). The experiment was repeated at least three times with three parallel samples. These results suggest that Ctri9594 may effectively inhibit the growth of Gram-positive bacteria but appears to have no inhibitory activity against Gram-negative bacteria or fungi.

## 3. Discussion

In this study, the antimicrobial peptide gene Ctri9594 was identified and cloned from the venom gland cDNA library of *Chaerilus tricostatus*, collected in Motuo of Tibet in China. *Chaerilus tricostatus* is a rare scorpion species and is only distributed in South Asia, Southeast Asia and southern Tibet in China. Because it has a limited distribution and has received little research attention, the scorpion *Chaerilus tricostatus* and its venom peptides have not been well characterized. *Chaerilus tricostatus* is also considered to be a scorpion species with no clinical significance after accidents. Therefore, to date, there are only six reports on the scorpion *Chaerilus tricostatus*, involving the venom gland cDNA construction, the antimicrobial peptide Ctriporin, the Kv1.3-blocking peptide Ctri9577 and the immunomodulating peptide Ctri18 [12,13,14,15]. However, some rare scorpion species may contain genetic information that is valuable to evolutionary and medical research.

The typical habitat of the scorpion *Chaerilus tricostatus* is under stones or fallen trees in humid or mesic regions. Such living environments are rich in diverse microorganisms, especially bacteria. With a long evolutionary history, this scorpion produces a variety of innate immune molecules, including antimicrobial peptides against these microorganisms to combat infection. In addition to predation and defense against natural enemies, the scorpion venom gland has evolved many antimicrobial peptides to prevent bacterial infections when stinging. The discovery of the antimicrobial peptide Ctri9577 from the venom of the scorpion *Chaerilus tricostatus* supports the conclusion that scorpion venom is rich in antimicrobial peptides.

Antimicrobial peptides are important effectors in the innate immune system [22]. Scorpion antimicrobial peptides were first found in the tissue of hemolymph, and then many AMPs were gradually isolated and identified from scorpion venoms. Currently, antimicrobial peptides isolated from scorpion venoms can be divided into three classes: scorpine-like peptides, long non-disulfide antimicrobial peptides and short non-disulfide antimicrobial peptides. In our study, the precursor nucleotide sequence of Ctri9594 was cloned and characterized from the venom cDNA library of the scorpion *Chaerilus tricostatus.* The Ctri9594 precursor nucleotide sequence was observed to contain an ORF with 69 amino acid residues, including a 21 aa signal peptide, 14 aa mature peptide, 3 aa C-terminal post-translational processing signal and 31 aa propeptide. The synthesized Ctri9594 mature peptide has inhibitory activity against Gram-positive bacteria, and its antimicrobial mechanism is related to its amphiphilic α-helix structure. From the perspective of structure and function, the antimicrobial peptide Ctri9594 from the venom of the scorpion *Chaerilus tricostatus* is a member of short non-disulfide antimicrobial peptides.

Antibiotic resistance has become a serious threat to global public health, which greatly increases the cost of treating bacterial infections [23]. The morbidity and mortality rates of antibiotic-resistant bacteria are significantly higher than those of antibiotic-sensitive strains. Over the past ten years, superbugs that are resistant to carbapenem, fluoroquinolones and even vancomycin have emerged. It is necessary to develop new antibacterial agents as alternatives to control and treat bacterial infections. As a class of potential candidates, antimicrobial peptides are used to combat antibiotic-resistant bacteria and have attracted people’s attention. With the increasing number of antimicrobial peptides and the improvement of artificial antimicrobial peptides, they are likely to be used as safe and effective new antimicrobial drugs in clinical practice, thus partially reducing or even replacing the use of traditional antibiotics [24,25].

Ctri9594 was revealed to be a typical amphiphilic cation α-helical peptide. Natural antimicrobial peptides with this structure are generally membrane-lytic peptides, which can kill bacteria in a few minutes [26]. These peptides bind to the bacterial membrane [27] and cause the leakage of intracellular components by nonspecifically increasing membrane permeability [28]. There are many negatively charged molecules on the outer surface of the bacterial membrane. The electrostatic attraction between the positively charged Ctri9594 peptide and the negatively charged bacterial surface promotes the binding of the peptide to the bacterial membrane [29]. Although antimicrobial peptides such as Ctri9594 have potential advantages over traditional antibiotics, such as low molecular mass, easy synthesis, high activity, good solubility, no residue in the body and no resistance, there are still some obstacles to their development as therapeutic drugs. For example, their rich hydrophobic amino acid residues lead to strong hemolysis and high cytotoxicity [30].

## 4. Materials and Methods

### 4.1. Screening cDNA Library with PCR Strategy

The venom gland cDNA library of the scorpion *Chaerilus tricostatus* was previously constructed [12,13]. The clones of this library were randomly selected from an LB plate and then cultured overnight in a liquid medium containing 35 μg/mL chloramphenicol in a 37 °C shaker. Using M13-positive and -negative primers of the library vector pDNR-LIB, colony PCR was performed to randomly screen cDNAs with 500 bp size.

### 4.2. cDNA Sequencing and Sequence Analysis

The plasmid pDNR-LIB with an approximately 500 bp-long insert fragment was determined by using the ABI 3730XL sequencer with a universal M13-positive primer. The linker sequence of pDNR-LIB was removed using CrossMatch. The open reading frame (ORF) and amino acid sequence were predicted by Generunr. The signal peptide sequence was analyzed using the SignalP 5.0 server (http://www.cbs.dtu.dk/services/SignalP-5.0/; accessed on: 01 April 2019). Homological sequences were searched with NCBI blast. Multiple sequence alignments were carried out using GeneDoc. The evolutionary relationship was inferred using the neighbor-joining method [19]. Evolutionary analysis was conducted in MEGA7 [21].

### 4.3. Chemical Synthesis

The peptide was chemically synthesized by GL Biochem (Shanghai, China). Since there is a processing signal sequence composed of three amino acid residues at the N-terminus of the C-terminal propeptide, the synthesized peptide was amidated at the C-terminus. RP-HPLC analysis was used to identify the purity of the synthetic peptide. The conditions of RP-HPLC were: C18 column, flow rate of 1 mL/min, mobile phase of 90% acetonitrile and 0.1% TFA and detection wavelength of 220 nm. Matrix-assisted laser desorption/ionization time-of-flight mass spectrometry (MALDI–TOF–MS) analysis was used to measure the average molecular mass of the synthetic peptide. A 1 μL aliquot of the peptide was spotted onto the target plate, along with an equal volume of a matrix solution (10 mg/mL α-cyano-4-hydroxycinnamic acid (CHCA), 50% acetonitrile and 0.1% TFA). The mixture was left to dry at room temperature. Mass spectrometry was performed using the FlexControl software (*m*/*z* range of 1000–5000).

### 4.4. Structure Prediction and CD Analysis

Secondary structure prediction of the mature peptide was performed by online analysis of the Heliquest website (https://heliquest.ipmc.cnrs.fr/cgi-bin/ComputParams.py; 01/07/2008). The synthetic Ctri9594 mature peptide was used to prepare sample solutions with a concentration of 0.1 mg/mL by dissolution in ultrapure water, 30% TFE and 70% TFE. The sample was added into a 2 mm diameter quartz cup, and the CD detection was then performed. The conditions of CD were: detection wavelength of 260–190 nm, scanning speed of 50 nm/min, scanning step of 1 nm and temperature of 25 °C. Each sample was scanned three times, and the average value of the three measurements was calculated as the final experimental data. The three-dimensional structure of the peptide was modeled using the website I-TASSER (https://zhanglab.dcmb.med.umich.edu/I-TASSER/; 10/01/2009).

### 4.5. Antimicrobial Assay

All antimicrobial assays were determined by following CLSI (the Clinical and Laboratory Standards Institute) guidelines. *Bacillus thuringensis* AB92037, *Bacillus subtilis* AB91021, *Staphylococcus aureus* AB94004, *Micrococcus luteus* AB93113, *Escherichia coli* AB94012, *Pseudomonas aeruginosa* AB93066 and *Candida albicans* AY93025 were purchased from China Center of Type Culture Collection (CCTCC). Minimal inhibitory concentration (MIC) was determined using a 96-well microtiter plate with a final volume of 100 μL containing microbes at a concentration of 10^4^ to 10^5^ colony-forming units per milliliter in LB culture medium. The synthetic peptide was added to obtain final concentrations of 100, 50, 25, 12.5, 6.25, 3, 1.5, 0.75 and 0 μg/mL. Each concentration was repeated three times. Ampicillin (Amp), Kanamycin (Kan) and Amphotericin B (Amb) were used as positive controls, and normal saline was used as a negative control. Inhibitory growth was examined by monitoring the absorbance at 630 nm (OD630) with a microplate reader after incubation at 37 °C for 12 h with continuous shaking.

## Figures and Tables

**Table 1:** Minimum inhibitory concentrations (MICs) of the chemically synthesized Ctri9594 mature peptide against bacterial strains.

Strains	MIC (μg/mL)			
	Ctri9594	Amp	Kan	Amb
Gram-positive bacteria				
*Bacillus thuringensis* AB92037	25	>100	4	ND
*Bacillus subtilis* AB91021	25	>100	20	ND
*Staphylococcus aureus* AB94004	25	2	50	ND
*Micrococcus luteus* AB93113	12.5	10	20	ND
Gram-negative bacteria				
*Escherichia coli* AB94012	>100	ND	60	ND
*Pseudomonas aeruginosa* AB93066	>100	ND	60	ND
Fungus				
*Candida albicans* AY93025	>100	ND	ND	0.6

Amp, Ampicillin. Kan, Kanamycin. Amb, Amphotericin B. ND, no determination.
